# Enzymatic degradation of sulfite-pulped softwoods and the role of LPMOs

**DOI:** 10.1186/s13068-017-0862-5

**Published:** 2017-07-11

**Authors:** Piotr Chylenski, Dejan M. Petrović, Gerdt Müller, Marie Dahlström, Oskar Bengtsson, Martin Lersch, Matti Siika-aho, Svein Jarle Horn, Vincent G. H. Eijsink

**Affiliations:** 1 Faculty of Chemistry, Biotechnology and Food Science, Norwegian University of Life Sciences, P.O. Box 5003, 1432 Ås, Norway; 2 Borregaard AS, P.O.Box 162, 1701 Sarpsborg, Norway; 3 VTT Technical Research Centre of Finland, P.O. Box 1000, 02044 VTT Espoo, Finland

**Keywords:** Lignocellulose, Sulfite, Pretreatment, Pulping, Cellulase, LPMO, AA9, GH61, Biofuel

## Abstract

**Background:**

Recent advances in the development of enzyme cocktails for degradation of lignocellulosic biomass, especially the discovery of lytic polysaccharide monooxygenases (LPMOs), have opened new perspectives for process design and optimization. Softwood biomass is an abundant resource in many parts of the world, including Scandinavia, but efficient pretreatment and subsequent enzymatic hydrolysis of softwoods are challenging. Sulfite pulping-based pretreatments, such as in the BALI™ process, yield substrates that are relatively easy to degrade. We have assessed how process conditions affect the efficiency of modern cellulase preparations in processing of such substrates.

**Results:**

We show that efficient degradation of sulfite-pulped softwoods with modern, LPMO-containing cellulase preparations requires the use of conditions that promote LPMO activity, notably the presence of molecular oxygen and sufficient reducing power. Under LPMO activity-promoting conditions, glucan conversion after 48-h incubation with Cellic^®^ CTec3 reached 73.7 and 84.3% for Norway spruce and loblolly pine, respectively, at an enzyme loading of 8 mg/g of glucan. The presence of free sulfite ions had a negative effect on hydrolysis efficiency. Lignosulfonates, produced from lignin during sulfite pretreatment, showed a potential to activate LPMOs. Spiking of Celluclast^®^, a cellulase cocktail with low LPMO activity, with monocomponent cellulases or an LPMO showed that the addition of the LPMO was clearly more beneficial than the addition of any classical cellulase. Addition of the LPMO in reactions with spruce increased the saccharification yield from approximately 60% to the levels obtained with Cellic^®^ CTec3.

**Conclusions:**

In this study, we have demonstrated the importance of LPMOs for efficient enzymatic degradation of sulfite-pulped softwood. We have also shown that to exploit the full potential of LPMO-rich cellulase preparations, conditions promoting LPMO activity, in particular the presence of oxygen and reducing equivalents are necessary, as is removal of residual sulfite from the pretreatment step. The use of lignosulfonates as reductants may reduce the costs related to the addition of small molecule reductants in sulfite pretreatment-based biorefineries.

**Electronic supplementary material:**

The online version of this article (doi:10.1186/s13068-017-0862-5) contains supplementary material, which is available to authorized users.

## Background

Enzymatic degradation of lignocellulose plays a pivotal role in a future biomass-based economy. Woody biomass is a sustainable source of lignocellulosic biomass in many parts of the world, including Scandinavian countries, North and South America, and New Zealand, and its efficient biochemical conversion is of major importance for the transition toward a biomass-based economy [1]. Recalcitrance of lignocellulosic biomass is one of the key factors hampering commercialization of plant biomass-derived fuels and chemicals and woody biomass is particularly challenging due to its physical properties, such as a dense structure and a high lignin content [1, 2]. To overcome biomass recalcitrance, various pretreatment technologies have been introduced. Pretreatments for woody biomass include steam explosion (usually acid-catalyzed), dilute acid, organosolv, and sulfite-pretreatment-to-overcome-recalcitrance-of-lignocellulose (SPORL). Generally, pretreatment and subsequent enzymatic digestion of hardwoods is less demanding compared to softwoods, which contain more lignin and have more rigid structure [1, 3]. Relatively efficient pretreatment and enzymatic hydrolysis of softwoods has been demonstrated for organosolv- and SPORL-pretreated biomasses [4, 5]. The BALI™ process is an alternative sulfite-based pretreatment that yields cellulose-rich substrates containing little lignin and hemicellulose relative to, e.g., the SPORL pretreatment, whose enzymatic conversion to glucose is relatively easy [6].

Complete biochemical conversion of cellulose to monomeric sugars requires the synergistic action of several classes of enzymes [7]. By far, the most studied enzymes are cellulose-degrading glycoside hydrolases, known as cellulases. Cellulases include enzymes acting on the ends of the cellulose chain, known as cellulose 1,4-β-cellobiosidases or cellobiohydrolases, cleaving off cellobiose units from reducing and non-reducing chain ends. Endo-β-1,4-glucanases cleave internal glycosidic bonds in the cellulose chains, thus generating novel chain ends, and β-glucosidases convert released soluble cellodextrins and cellobiose to glucose [8]. In natural biomass-converting enzyme systems, the cellulases are accompanied by a wide range of hemicellulose- and lignin-degrading enzymes working in concert to fully decompose lignocellulose.

The discovery of lytic polysaccharide monooxygenases (LPMOs) has challenged the classical model of enzymatic decomposition of polysaccharides. Contrary to cellulases, which rely on hydrolysis to perform catalysis, LPMOs are metalloenzymes breaking down glycosidic bonds by an oxidative mechanism involving molecular oxygen (or, in a recently suggested alternative mechanism, hydrogen peroxide [9]) and an electron donor [10, 11]. LPMO-catalyzed cleavage of the glycosidic bond results in the formation of oxidized chain ends, either at the C1 and/or at the C4 carbon position. So far, LPMOs cleaving glycosidic bonds in chitin [10, 12], cellulose [11, 13–16], cello-oligosaccharides [17], hemicellulose [18, 19], and starch [20, 21] have been identified. LPMOs are classified in the Carbohydrate Active Enzymes (CAZy) database as auxiliary activities families AA9, AA10, AA11, and AA13 [22].

Despite the fact that the boosting effect of LPMOs on cellulase activity was demonstrated early on [13, 15], exploration of the role and performance of LPMOs in applied settings has been limited. Cannella et al. [23] showed that LPMOs present in the commercial cellulase preparation Cellic^®^ CTec2 enhanced hydrolysis of hydrothermally pretreated wheat straw. Müller and colleagues showed the importance of LPMOs in this enzyme preparation for degradation of steam-exploded birch [24]. Using both model cellulosic substrates and industrially relevant lignocellulosic biomass, Hu et al. [25] demonstrated synergy between AA9 LPMOs and cellulases.

In this study, we have investigated the impact of process conditions on the enzymatic degradation of sulfite-pulped softwood biomasses with particular focus on the role of LPMOs, the presence of oxygen and small molecule electron donors, and the effect of residual sulfite. We have utilized commercially available cellulase preparations, Celluclast^®^ 1.5L (supplemented with Novozym 188) and Cellic^®^ CTec3, as well as an in-house produced fungal LPMO from *Thermoascus aurantiacus* (*Ta*LPMO9A) and individual major *Trichoderma reesei* cellulases.

## Methods

### Cellulosic substrates, pretreatment, and compositional analysis

Norway spruce (*Picea abies*) and loblolly pine (*Pinus taeda*) were subjected to the proprietary pretreatment technology developed by Borregaard AS (Sarpsborg, Norway) [6, 26]. The pretreatment included a sulfite cooking step utilizing calcium or sodium as a counterion, which converts lignin into water-soluble lignosulfonates and removes most of the hemicellulose that is washed out of the remaining cellulose pulp. The composition of the pretreated materials was determined following the National Renewable Energy Laboratory standardized protocol (NREL/TP-510-42618) and is presented in Table 1. The commercially available model cellulosic substrate Avicel PH-101 (Sigma Aldrich, St. Louis, MO, USA) was also used in enzymatic hydrolysis experiments. Dried lignosulfonates were kindly provided by Borregaard AS (Norway, Sarpsborg) and were obtained by the evaporation of the sulfite spent liquor (SSL) that had been stripped of soluble sugars by fermentation [6].

**Table 1:** Compositional analysis of pretreated Norway spruce and Loblolly pine

Substrate	Component (% of DM)^a^			
	Glucan^b^	Xylan^b^	Mannan^b^	Acid insoluble lignin^c^
Norway spruce	88.3	4.5	4.8	3.8
Loblolly pine	80.4	3.0	3.0	8.9
Avicel	92.2	2.1	0.1	0.9

Values are presented as a percentage of dry matter

^a^The presence of the potentially inhibitory sugar derivatives furfural and hydroxymethylfurfural was also analyzed; both were not detected

^b^Sugar monomers measured by HPLC and corrected by the hydration factor (*0.9)

^c^Klason lignin (not corrected for ash)

### Enzymes

Celluclast^®^ 1.5L, Novozym 188, and Cellic^®^ CTec3 were all kindly provided by Novozymes A/S (Bagsværd, Denmark). The AA9 family LPMO from *Neurospora crassa* (*Nc*LPMO9C), used for generation of C4 oxidized standards, was produced and purified as described previously [24]. *Trichoderma reesei* Cel7A was purified from the culture filtrate of *T. reesei* QM 9414 (VTT Culture Collection, D-74075, Finland) essentially as described in [27]. *Tr*Cel7B and *Tr*Cel6A from *T. reesei* were purified as described by Suurnäkki et al. [28].

The gene encoding *T. aurantiacus* LPMO9A (also known as *Ta*GH61A; [11]) including its native signal sequence was codon optimized for *Pichia pastoris* (GenScript, Piscataway, NJ, USA). A synthetic, *Pichia*-optimized gene encoding *Ta*LPMO9A was excised from the pUC57 vector using *Acc*65I and *Eco*RI restriction enzymes (New England BioLabs [NEB] Inc, MA, USA) and ligated into the same sites of the pPINK-GAP vector [29], yielding the pPINK-GAP_*Ta*LPMO9A plasmid. This plasmid was then linearized with *AflII* and 5 μg of linearized plasmid was transformed into freshly prepared electrocompetent *P. pastoris* PichiaPink™ Strain 4 cells, following the manufacturer’s instructions (Thermo Fisher Scientific, Waltham, MA, USA), using a Bio-Rad Gene Pulser II electroporation system (Bio-Rad Laboratories, Hercules, CA, USA) at 1.8 kV, 25 μF, and 200 Ω. Subsequently, the cells were incubated in 1 mL of YPDS medium for 2 h and spread on *Pichia* adenine dropout (PAD) selection plates (Thermo Fisher Scientific, Waltham, MA, USA), followed by incubation for 3–4 days at 30 °C, until white colonies formed. Four colonies were picked and restreaked on fresh PAD plates. Overnight cultures of these four transformants were then screened for protein production in BMGY medium (containing 1% (v/v) glycerol). A glycerol stock was prepared from an overnight culture of the best-producing transformant in BMGY medium by adjusting the cell suspension to 28% (v/v) glycerol.

The best-producing transformant was grown in 20 mL of BMGY medium (containing 1% (v/v) glycerol) in a 100-mL shaken flask at 29 °C and 200 rpm for 16 h. Subsequently, this pre-culture was used to inoculate 4 × 0.5 L of BMGY medium (containing 1% (v/v) glycerol) in 2-L shaken flasks, followed by incubation at 29 °C and 200 rpm for 48 h. After 24 h, the cultures were supplemented with 1% (v/v) glycerol. The cells were harvested by centrifugation at 7.000*g* for 15 min, at 4 °C. The supernatants were collected and dialyzed against 50 mM Bis–Tris buffer, pH 6.5, and concentrated to 150 mL using a VivaFlow 200 tangential crossflow concentrator (MWCO 10 kDa, Sartorius Stedim Biotech Gmbh, Germany). Ammonium sulfate was added to the concentrated supernatant to a final concentration of 1.42 M after which the solution was loaded onto three linearly connected 5-mL HiTrap Phenyl FF columns (GE Healthcare Life Sciences, Uppsala, Sweden) equilibrated with 50 mM Bis–Tris buffer (pH 6.5), containing 1.42 M ammonium sulfate. Proteins bound to the columns were eluted using a 75 mL linear gradient from 1.42 to 0 M ammonium sulfate in 50 mM Bis–Tris buffer (pH 6.5), using a flow rate of 1 mL/min. Collected fractions were analyzed by sodium dodecyl sulfate polyacrylamide gel electrophoresis (SDS-PAGE), and the fractions containing *Ta*LPMO9A were pooled and subsequently concentrated and buffer exchanged to 50 mM Bis–Tris pH 6.5 using Amicon Ultra centrifugal filters (MWCO 10 kDa, Millipore).

Purified *Ta*LPMO9A was saturated with Cu (II) by incubating the enzyme with an excess of CuSO_4_ (3:1 molar ratio of copper:enzyme) for 90 min at room temperature as described previously [30]. Subsequently, the protein solution was loaded onto a HiLoad 16/60 Superdex 75 size exclusion column (GE Healthcare Life Sciences, Uppsala, Sweden) in 50 mM Bis–Tris buffer (pH 6.5), containing 150 mM NaCl, using a flow rate of 0.75 mL/min. Fractions containing pure protein were identified using SDS-PAGE and subsequently pooled and concentrated using Amicon Ultra centrifugal filters (MWCO 10 kDa, Millipore).

To ensure full copper saturation, the purified LPMO was saturated once more with Cu (II), as described above. This time, after incubating the enzyme with CuSO_4_, the solution was loaded onto a PD midiTrap G-25 desalting column (GE Healthcare, UK), equilibrated with 20 mM Bis–Tris buffer pH 6.0. Protein-containing fractions, eluted with 1 mL of equilibration buffer, were collected and stored at 4 °C until further use.

All protein concentrations were determined with the Bio-Rad modified Bradford method (Bio-Rad Laboratories, Hercules, CA, USA) utilizing bovine serum albumin (BSA) as a standard [31].

### Enzymatic hydrolysis

Saccharification of lignocellulosic biomass and Avicel was conducted in 50-mL rubber sealed glass bottles (Wheaton, Millville, NJ, USA) with 10 mL working volume. The biomass obtained after the sulfite pulping process described above was not washed, milled, or dried prior to the saccharification experiments. Enzymatic hydrolysis was performed with 5% total solids loading in 50 mM sodium acetate buffer pH 5.0 at 50 °C, with 8 mg/g glucan total protein loading of either a 5:1 (w/w) Celluclast^®^:Novozym 188 mixture or Cellic^®^ CTec3, in the presence or absence of an external electron donor. To facilitate efficient mixing, bottles were rotated at 38 rpm in a Multi RS-60 programmable rotator (Biosan, Riga, Latvia). Anaerobic conditions were reached by vigorously flushing the substrate-buffer suspension with nitrogen (Yara, Trondheim, Norway) for 3 min and addition of 0.025% (w/v, final concentration) of l-cysteine hydrochloride monohydrate (Sigma Aldrich, St. Louis, MO, USA) to ensure complete removal of oxygen. In aerobic conditions, oxygen came from ambient air present in the headspace of the reaction bottles. Reactions were initiated by injection of 800 µL of enzyme preparations, appropriately diluted in 50 mM sodium acetate buffer pH 5.0, through the rubber septum. Reactions were terminated at different time points. To ensure reproducible sampling from the flasks, the entire reaction mixture was diluted threefold with ultrapure water (Merck Millipore, Billerica, MA, USA) and then transferred to new 50-mL Falcon tubes. Reactions were stopped by incubating the Falcon tubes at 100 °C for 15 min in a water bath. Supernatants were collected by centrifugation of the tubes for 15 min at 3803*g* and 4 °C, and these were then transferred to 1.5-mL tubes and stored at −20 °C prior to further analysis.

### Product analysis

Glucose and cellobiose released during enzymatic hydrolysis were quantified with High-Performance Liquid Chromatography (HPLC) using a Dionex Ultimate 3000 system (Dionex, Sunnyvale, CA, USA) coupled to a refractive index (RI) detector 101 (Shodex, Tokyo, Japan). Separation of hydrolysis products was achieved utilizing a Rezex ROA-Organic Acid H^+^ (8%), 300 × 7.8 mm analytical column equipped with SecureGuard Carbo-H^+^ 4 × 3.0 mm guard column (Phenomenex, Torrance, CA, USA), operated at 65 °C, with 5 mm H_2_SO_4_ as the mobile phase, and a flow rate of 0.6 mL/min. For quantification, the areas of peaks corresponding to glucose and cellobiose were compared to standard curves generated with known concentrations of glucose and cellobiose (in the range of 0.1–10 g/L). Hydrolysis yields were calculated based on detected glucose and cellobiose (typically less than 1% of the total) and expressed as a percentage of the theoretical maximum that would be obtained upon complete conversion of glucan to glucose.

Statistical significance of differences in glucan saccharification yields was determined using two-way ANOVA with Tukey’s post hoc test (95% confidence interval) and was carried out using R (R Foundation for Statistical Computing, Vienna, Austria). Statistical significance is shown as follows: *p < 0.05, **p < 0.01, ***p < 0.001.

Native cello-oligosaccharides (DP 2 to DP 5) and oxidized sugars were analyzed with High-Performance Anion Exchange Chromatography with Pulsed Amperometric Detection (HPAEC-PAD) using a Dionex ICS 3000 system (Dionex, Sunnyvale, CA, USA) equipped with a CarboPac PA1 2 × 250 mm analytical column with a CarboPac PA1 2 × 50 mm guard column, as described in [15]. Briefly, initial conditions were set to 0.1 M NaOH (100% eluent A), followed by a linear gradient of eluent B (1 M sodium acetate in 0.1 M NaOH), reaching 10% B 10 min after sample injection and 30% B at 35 min after injection. This was followed by a 5-min exponential gradient to 100% eluent B, after which the column was reconditioned by running the initial conditions for 9 min.

C4-oxidized standards were generated with *Nc*LPMO9C by cellopentaose degradation as described in [24]. The data were collected and analyzed using Chromeleon 7.0 software.

## Results

Below, several series of hydrolysis experiments are described. Table 2 provides an overview of the hydrolysis yields obtained in these experiments.

**Table 2:** Overview of hydrolysis yields for various degradation reactions

Raw material	Enzyme	Conditions	AscA	Sulfite (12.5 mM)	Glucan solubilization after 48 h (% of theoretical)
Avicel PH-101	Cellic^®^ CTec3	Aerobic	+	−	69.0 ± 1.9
		Aerobic	+	+	31.6 ± 0.4
		Anaerobic	+	−	50.0 ± 1.4
		Anaerobic	+	+	44.1 ± 1.2
	Celluclast^®^:Novozym 188	Aerobic	+	−	55.6 ± 0.9
		Aerobic	+	+	39.5 ± 0.5
		Anaerobic	+	−	57.1 ± 0.7
		Anaerobic	+	+	38.6 ± 4.3
Norway spruce	Cellic^®^ CTec3	Aerobic	+	−	73.7 ± 0.7
		Aerobic	+	+	34.1 ± 0.4
		Anaerobic	+	−	50.6 ± 3.8
		Anaerobic	+	+	55.6 ± 2.3
	Celluclast^®^:Novozym 188	Aerobic	+	−	64.9 ± 2.1
		Aerobic	+	+	38.7 ± 1.5
		Anaerobic	+	−	60.2 ± 1.4
		Anaerobic	+	+	50.3 ± 2.6
Loblolly pine	Cellic^®^ CTec3	Aerobic	+	−	84.3 ± 1.2
		Anaerobic	+	−	60.3 ± 0.7

### Saccharification of a model cellulosic substrate

**Fig. 1:**
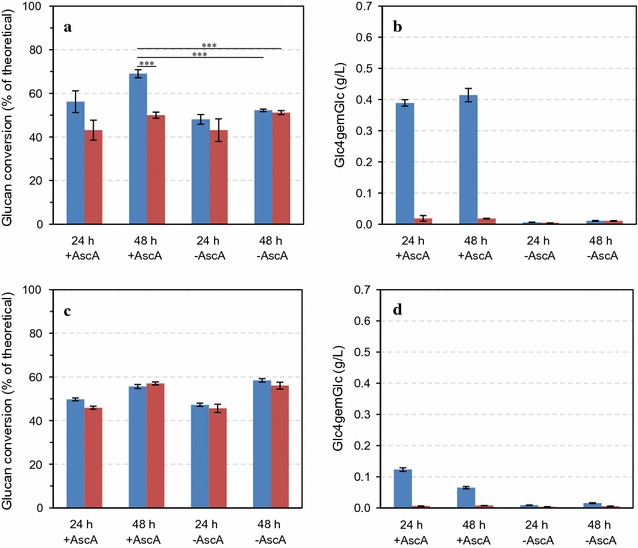
Saccharification of Avicel PH-101 under aerobic (*blue bars*) or anaerobic (*red bars*) conditions in the presence or absence of 1 mM ascorbic acid (±AscA). The *left panels* show glucan conversion (as a percentage of theoretical glucan conversion) and the *right panels* show the concentration of Glc4gemGlc, at two time points. Enzymatic hydrolysis was carried out with Cellic^®^ CTec3 (**a**, **b**) and Celluclast^®^:Novozym 188 (mixed at 5:1 ratio, w/w) (**c**, **d**) at 8 mg/g glucan total protein loading, using reaction mixtures containing 5% DM in 50 mM sodium acetate pH 5.0 that were incubated at 50 °C. The data points represent the average value of three independent experiments with one technical replicate per experiment. The *error bars* represent standard deviations of the three independent experiments. The statistical significance of differences in the 48-h saccharification yields was analyzed using two-way ANOVA with Tukey’s post hoc test (95% confidence interval) and is indicated as follows: *p < 0.05, **p < 0.01, ***p < 0.001

The model cellulosic substrate Avicel PH-101 was incubated for 48 h with an older generation, LPMO-poor, cellulase mixture (Celluclast^®^ 1.5L supplemented with Novozym 188; 5:1, w/w), as well as a modern, LPMO-containing, cellulase preparation, Cellic^®^ CTec3, under either aerobic or anaerobic conditions, in the presence or absence of an external electron donor (ascorbic acid). These experiments showed that Cellic^®^ CTec3 gives higher hydrolysis yields, but only under aerobic conditions and when adding an external electron donor (Fig. 1a). Indeed, under these conditions only, considerable LPMO activity was detectable (Fig. 1b; the amount of C4-oxidized product amounts to approximately 1.5% of the amount of solubilized glucan). Saccharification of Avicel with the Celluclast^®^ 1.5L:Novozym 188 mixture was hardly affected by the presence of oxygen or the addition of ascorbic acid (Fig. 1c), and under LPMO-promoting conditions, only low product levels were observed (Fig. 1d). Celluclast^®^ 1.5L is a product based on the *T. reesei* secretome, in which three AA9 family LPMOs have been identified, which however, are expressed at low levels [13, 32].

### Saccharification of sulfite-pulped softwoods

**Fig. 2:**
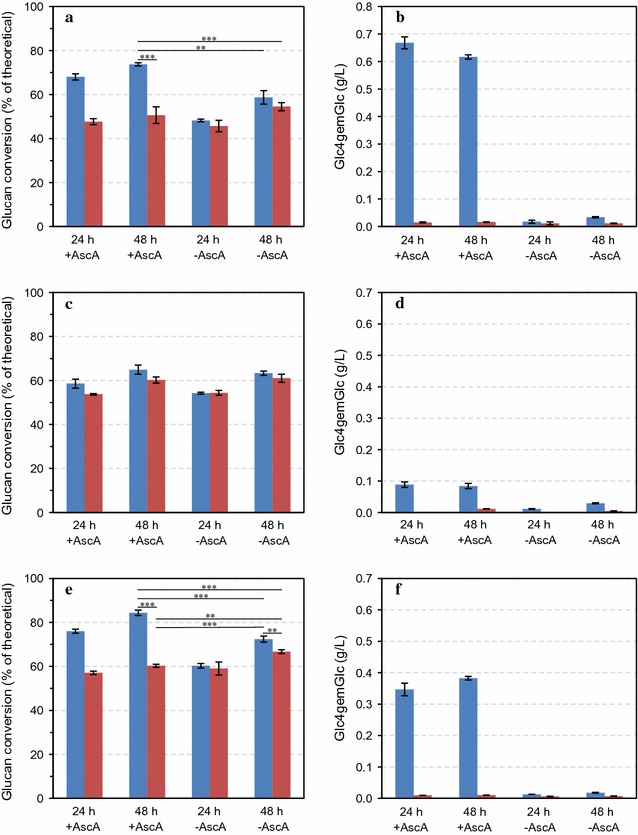
Saccharification of pretreated Norway spruce (**a**–**d**) and loblolly pine (**e**–**f**) under aerobic (*blue bars*) or anaerobic (*red bars*) conditions in the presence or absence of 1 mM ascorbic acid (±AscA). The *left panels* show glucan conversion (as a percentage of theoretical glucan conversion) and the *right panels* show the concentration of Glc4gemGlc, at two time points. Enzymatic hydrolysis was carried out with Cellic^®^ CTec3 (**a**, **b**, **e**, **f**) and Celluclast^®^:Novozym 188 (mixed at 5:1 ratio, w/w) (**c**, **d**) at 8 mg/g glucan total protein loading using reaction mixtures containing 5% DM in 50 mM sodium acetate pH 5.0 that were incubated at 50 °C. The data points represent the average value of three independent experiments with one technical replicate per experiment. The *error bars* represent standard deviations of the three independent experiments. The statistical significance of differences in the 48-h saccharification yields was analyzed using two-way ANOVA with Tukey’s post hoc test (95% confidence interval) and is indicated as follows: *p < 0.05, **p < 0.01, ***p < 0.001

Sulfite-pulped Norway spruce was incubated with both cellulase preparations for 48 h, under conditions identical to those used for Avicel, with strikingly similar results (Fig. 2a–d). Again, Cellic^®^ CTec3 was the most efficient and, again, only under aerobic conditions and when adding an external source of electrons (Fig. 2a). Saccharification of pretreated Norway spruce with the LPMO-poor cocktail was not improved by aerobic conditions or the presence of electron donor (Fig. 2c). Oxidized products were only observed for reactions with oxygen and externally added electron donor present, and the levels of oxidized product were much higher for Cellic^®^ CTec3 (Fig. 2b), compared to the Celluclast^®^ reaction (Fig. 2d). This result shows that the heavily delignified sulfite-pretreated softwood biomass lacks the potential to activate LPMOs.

Saccharification of sulfite-pulped loblolly pine with Cellic^®^ CTec3 using the same conditions and varying the same parameters showed the same trends as observed for Norway spruce and Avicel. Interestingly, saccharification of pretreated loblolly pine was clearly more efficient than identically pretreated Norway spruce (compare Fig. 2e with a). The higher conversion of loblolly pine was achieved despite a seemingly lower LPMO activity (compare Fig. 2f with b).

**Fig. 3:**
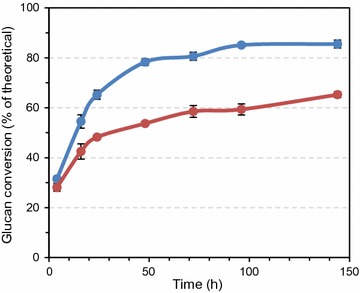
Saccharification of pretreated Norway spruce under aerobic (*blue line*) or anaerobic (*red line*) conditions in the presence of 1 mM ascorbic acid. Enzymatic hydrolysis was carried out with Cellic^®^ CTec3 at 8 mg/g glucan total protein loading in reaction mixtures containing 5% DM in 50 mM sodium acetate pH 5.0 that were incubated at 50 °C. The data points represent the average value of three independent experiments with one technical replicate per experiment. The *error bars* represent standard deviations of the three independent experiments

The importance of oxygen, which affects LPMO activity, was explored further using extended reaction times (up to 144 h). The results showed that enzymatic hydrolysis of sulfite-pulped Norway spruce under anaerobic conditions never reached the yields obtained under conditions promoting LPMO activity, the final yields being about 65 and 85%, respectively (Fig. 3).

Reduced glutathione and gallic acid can also drive LPMO activity by acting as electron donors [10, 15, 25]. Similarly to the addition of ascorbic acid, addition of each of these reducing agents improved enzymatic degradation of pretreated Norway spruce under aerobic conditions (Additional file 1: Figure S1a, b) albeit less efficiently than ascorbic acid (Fig. 2a).

Repeating the experiments with sulfite-pulped Norway spruce, in the absence of l-cysteine hydrochloride monohydrate (0.025%, w/v), showed that the addition of this compound, used to ensure anaerobic conditions, had no effect on hydrolysis yields (Additional file 2: Figure S2).

### Effect of residual sulfite on softwood saccharification

**Fig. 4:**
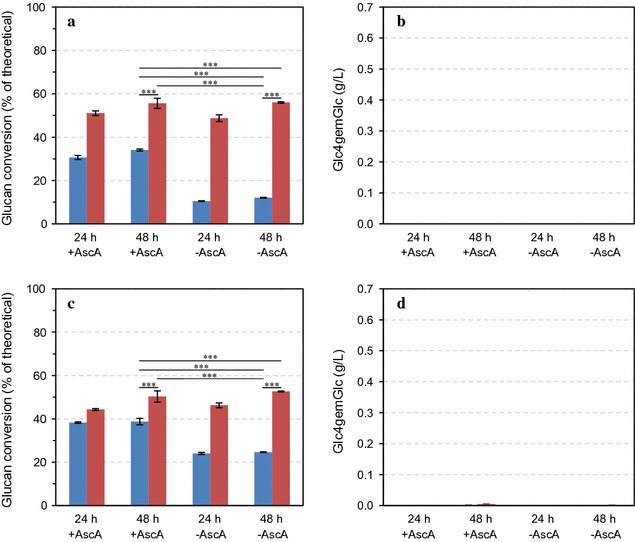
Saccharification of pretreated Norway spruce in the presence of sulfite under aerobic (*blue bars*) or anaerobic (*red bars*) conditions, in the presence or absence of 1 mM ascorbic acid (±AscA). The *left panels* show glucan conversion (as a percentage of theoretical glucan conversion) and the *right panels* show concentrations of Glc4gemGlc, at two time points. Enzymatic hydrolysis was carried out with Cellic^®^ CTec3 (**a**, **b**) and Celluclast^®^:Novozym 188 (mixed at 5:1 ratio, w/w) (**c**, **d**) at 8 mg/g glucan total protein loading in reactions containing 5% DM in 50 mM sodium acetate pH 5.0 that were incubated at 50 °C. Data for similar reactions without sulfite are presented in Fig. 2 and data for the effect of sulfite on hydrolysis of Avicel are presented in Additional file 3: Figure S3. The data points represent the average value of three independent experiments with one technical replicate per experiment. The *error bars* represent standard deviations of the three independent experiments. The statistical significance of differences in the 48-h saccharification yields was analyzed using two-way ANOVA with Tukey’s post hoc test (95% confidence interval) and is indicated as follows: *p < 0.05, **p < 0.01, ***p < 0.001

The sulfite-pretreated biomasses used in the experiments described above had been stored for some time and were likely devoid of residual sulfite, which is readily oxidized to sulfate in a reaction that involves oxygen and leads to the generation of various reactive intermediate species [33]. Sulfite pretreatment of lignocelluloses in a factory may result in residual sulfite still being present in the material that is subjected to subsequent enzymatic hydrolysis. To test how residual sulfite would affect enzymatic hydrolysis, we studied the effect of sulfite on hydrolysis of Avicel (Additional file 3: Figure S3) and pretreated Norway spruce (Fig. 4) by Cellic^®^ CTec3 and the Celluclast^®^-based cocktail. We tested the effect of adding 1000 ppm (12.5 mM) of sulfite ions (in the form of freshly added sodium sulfite), which was considered to be at the higher end of the concentrations that could be expected to be present in freshly pretreated and washed biomass, based on process data from the BALI™ pilot plant at Borregaard AS. Generally, sulfite had a negative effect on enzymatic hydrolysis of all substrates and no LPMO products could be detected (Fig. 4; see also Additional file 1: Figure S1, Additional file 2: Figure S2, Additional file 3: Figure S3). Maximal conversion with Cellic^®^ CTec3 (i.e., aerobic, with AscA, 48 h) was reduced from 69.0 and 73.7% to 31.6 and 34.1% for Avicel and Norway spruce, respectively (Table 2). These decreases in yields are larger than the effects of oxygen and ascorbic acid discussed above and are thus not likely to be only “LPMO effects.” Indeed, the efficiency of the LPMO-poor Celluclast^®^ mixture was also affected by sulfite, albeit to a lesser extent (Table 2; Fig. 4; Additional file 3: Figure S3). The effect of sulfite was clearly less pronounced under anaerobic conditions, which could be due to the absence of reactions between oxygen and sulfite that may lead to the formation of various compounds, including the sulfite radical and other radicals [33] that inhibit the enzymes. In line with this, the addition of reducing agent (or “antioxidant”) to the aerobic reactions had a clear positive effect on yields, despite the absence of LPMO activity (e.g., Figure 4). In the absence of reducing agent and in the presence of sulfite, hydrolysis yields were generally very low.

### Effect of lignosulfonates on degradation of sulfite-pretreated softwoods

**Fig. 5:**
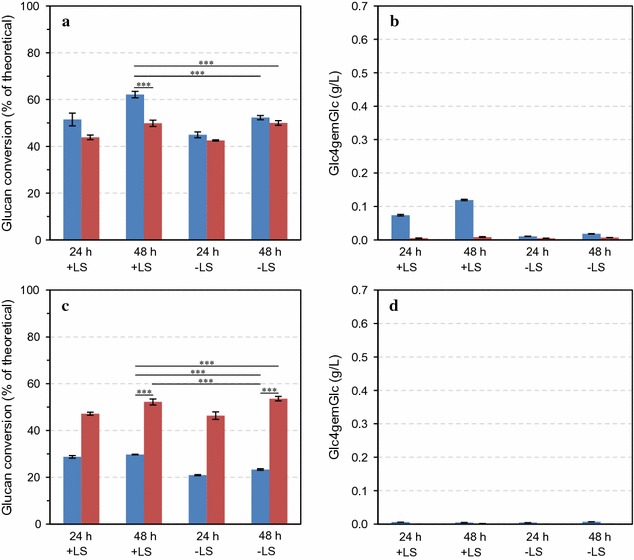
Saccharification of pretreated Norway spruce in the absence (**a**, **b**) or presence (**c**, **d**) of sulfite under aerobic (*blue bars*) or anaerobic (*red bars*) conditions, in the presence or absence of 0.25% (w/w) lignosulfonates (LS). The *left panels* show glucan conversion (as a percentage of theoretical glucan conversion) and the *right panels* show concentrations of Glc4gemGlc, at two time points. Enzymatic hydrolysis was carried out with Cellic^®^ CTec3 at 8 mg/g glucan total protein loading in reactions containing 5% DM in 50 mM sodium acetate pH 5.0 that were incubated at 50 °C. The data points represent the average value of three independent experiments with one technical replicate per experiment. The *error bars* represent standard deviations of the three independent experiments. The statistical significance of differences in the 48-h saccharification yields was analyzed using two-way ANOVA with Tukey’s post hoc test (95% confidence interval) and is indicated as follows: *p < 0.05, **p < 0.01, ***p < 0.001

Besides a cellulose-rich pulp, sulfite pretreatment also yields lignosulfonates (LS), representing a valuable commodity from the lignin fraction of softwood biomass. It has been shown that LS can improve enzymatic saccharification of pretreated lignocellulosic materials [34, 35]. Using enzymatic hydrolysis of sulfite-pulped Norway spruce as a test case, we assessed whether LS could replace ascorbic acid. It can be observed from Fig. 5 that 0.25% (w/w) LS led to increased hydrolysis yields under aerobic conditions, but to a smaller extent than the addition of ascorbic acid (Fig. 2). The increase in yield obtained by adding LS was accompanied by the production of oxidized products (Fig. 5b), albeit, again, at lower levels compared to reactions with ascorbic acid (Fig. 2b). Addition of LS helped also to mitigate the negative effect of sulfite ions on hydrolysis efficiency (Fig. 5c), but to a lower extent than ascorbic acid (Fig. 4a) and other tested reducing agents (Additional file 1: Figure S1c, d).

### Contribution of individual enzymes to saccharification efficiency

**Fig. 6:**
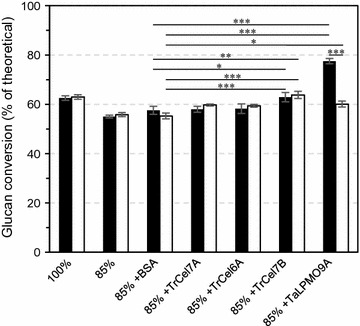
Saccharification of pretreated Norway spruce in the presence (*black bars*) or absence (*white bars*) of 1 mM ascorbic acid. Enzymatic hydrolysis was carried out with the Celluclast^®^:Novozym 188 mix (5: 1 w/w) supplemented with major *Trichoderma reesei* cellulases (*Tr*Cel7A, *Tr*Cel6A, and *Tr*Cel7B) or *Ta*LPMO9A. The total protein loading was 8 mg/g glucan, except for the sample labeled “85%,” where the total protein loading was at 6.8 mg/g glucan. The indicated individual enzymes comprised 15% of the total protein loading; BSA was used as a control. Experiments were carried out in reactions containing 5% DM in 50 mM sodium acetate pH 5.0 at 50 °C and were incubated at 50 °C for 48 h. The data points represent the average value of three independent experiments with one technical replicate per experiment. The *error bars* represent standard deviations of the three independent experiments. The statistical significance of differences in glucan conversion between the control reactions with BSA and reactions with added individual enzymes (in the presence or absence of 1 mM ascorbic acid) was analyzed using two-way ANOVA with Tukey’s post hoc test (95% confidence interval) and is indicated as follows: *p < 0.05, **p < 0.01, ***p < 0.001

Previous work on the development of minimal enzyme cocktails for conversion of BALI™-pretreated spruce has shown that of the best known individual *T. reesei* cellulases, *Tr*Cel7B is the most important [36]. This study also indicated that a cellulose-active, bacterial LPMO (*Sc*LPMO10C) had little effect on overall saccharification efficiency. Considering these previous results and the clear positive effect of LPMOs contained in Cellic^®^ CTec3 on the saccharification of sulfite-pulped softwoods, we tested the potential of *Ta*LPMO9A and three major *T. reesei* cellulases (*Tr*Cel7A, *Tr*Cel7B, and *Tr*Cel6A) to improve the efficiency of the Celluclast^®^ 1.5L:Novozym 188 mix. We replaced 15% of the enzyme mixture by one of these individual enzymes because previous studies on spiking Celluclast^®^ with *Ta*LPMO9A had indicated that this level of replacement was optimal in terms of increased saccharification yield [24]. Replacement of 15% of total protein by individual cellulases revealed that the addition of *Tr*Cel7B was more beneficial than that of the other individual cellulases and led to a yield comparable with that obtained with 100% of the original cellulase preparation. In line with other results reported in this study, replacement of 15% of the cellulase preparation with *Ta*LPMO9A had a clear beneficial effect on glucan conversion, but only when also ascorbic acid was added (Fig. 6).

## Discussion

Since the discovery of the true nature of LPMOs [10], their role in the degradation of cellulose and pretreated lignocellulosic biomasses has been the subject of several studies [13, 23–25, 37]. For example, it has been shown that harnessing LPMO activity improves the saccharification of hydrothermally pretreated wheat straw [23] and steam-exploded birch [24] with an LPMO-rich cellulase preparation, Cellic^®^ CTec2. Both these studies also showed that LPMO activity does not require the presence of externally added small molecule reducing agents, as long as the substrate is rich in lignin. A similar conclusion was drawn by Hu et al. [25] in their study focused on spiking cellulase preparations with an AA9 LPMO in the degradation of several pretreated lignocelluloses. Importantly, the study by Müller et al. (2015) established the importance of the presence of oxygen in harnessing the full potential of LPMOs.

From previous work, it is known that sulfite-pretreated softwoods can be degraded quite efficiently with commercially available cellulase preparations [5, 38]. Sulfite pulping using the BALI™ pretreatment yields cellulose-rich and almost lignin-free materials that are also relatively easy to degrade [6]. So far, the potential role of LPMOs in the degradation of sulfite-pretreated biomasses has not been addressed, nor have the implications of a possible role of LPMOs on process design. The present experiments, using both a model cellulosic substrate and industrially relevant, sulfite-pulped softwoods, shed light on these issues. The data show a clear correlation between the efficiency of saccharification and the detection of LPMO-generated products, underpinning the major contribution of LPMOs to the saccharification process, which amounts to increases in saccharification yield by up to 24% (in the case of sulfite-pulped loblolly pine). The presence of an externally added electron donor and aerobic conditions during enzymatic hydrolysis are both crucial for LPMO activity and for fully exploiting the cellulolytic potential of modern cellulase preparations. The requirement for an external electron donor to harness LPMO activity separates sulfite-pulped lignocelluloses from biomasses subjected to other physicochemical pretreatments and is likely due to extensive lignin removal during sulfite pulping (Table 1). The small amount of remaining lignin bound to the cellulose is most likely sulfonated, which will alter its properties, e.g., the degree of hydrophilicity, relative to, e.g., the lignin present in steam-exploded materials [3]. Dimarogona et al. [39] and Westereng et al. [40] have demonstrated that lignin is capable of transferring electrons to LPMOs and stimulate their activity in cellulose decomposition, whereas Hu et al. [25] have shown that complete lignin removal from steam-pretreated lodgepole pine nearly completely eliminated the boosting effect of adding an LPMO. Finally, by studying enzymatic saccharification of sugarcane bagasse samples obtained from different pretreatments and with varying degrees of delignification, Rodríguez-Zúñiga et al. [41] showed a positive correlation between lignin content and LPMO activity. All these results confirm early observations by Harris et al. [13], who showed that the enigmatic boosting effect of a GH61 protein (which later turned out to be an LPMO) on cellulase activity depended on the presence of lignin.

In the presence of ascorbate and under aerobic conditions, improvement of the enzymatic degradation of sulfite-pretreated softwoods was correlated with the accumulation of oxidized products. The positive effect of LPMO-promoting conditions was only observed when using LPMO-rich cellulase preparation Cellic^®^ CTec3 and when spiking an older cellulase preparation, LPMO-poor Celluclast^®^ 1.5L, with *Ta*LPMO9A. The dependence on oxygen is evident from both the present study and earlier work on steam-exploded birch by Müller et al. [24]. Interestingly, there are recent indications that LPMO reactions may be driven by H_2_O_2_ instead of O_2_ and that in such a set-up the LPMO requires only substoichiometric amounts of reductant [9]. While further work is needed to study the implications of this novel way to drive LPMO activity, it is worth noting that the use of H_2_O_2_ may change or even abolish the need for supplying molecular oxygen and reductant in industrial biorefining. Supplying and controlling hydrogen peroxide, which is soluble in water, in high dry matter hydrolysis reactors may be easier, compared to using oxygen.

Careful inspection of Figs. 1 (hydrolysis of Avicel) and 2 (hydrolysis of sulfite-pulped Norway spruce) shows that under conditions that do not lead to LPMO activity, the Celluclast^®^:Novozym 188 cocktail is more efficient than Cellic^®^ CTec3. Under these conditions, the LPMO fraction of the Cellic^®^ CTec3 enzyme cocktail, perhaps amounting to 15% of the protein, is not active, meaning a reduced effective enzyme load. This observation underpins the importance of harnessing the power of LPMOs in modern cellulase cocktails.

While sulfite pulping generally seems to be a good pretreatment for softwoods, the present data show that residual sulfite that might be carried over from the pretreatment step to the enzymatic hydrolysis step has a negative effect on saccharification efficiency. The oxidation of sulfite to sulfate happens via a multi-step pathway involving the formation of several reactive intermediates [33] that are known to interact with, e.g., proteins [33, 42, 43]. For example, radicals formed during oxidation of sulfite react with methionine and tryptophan in proteins, which could easily lead to enzyme inactivation [33, 43]. Reducing agents, such as ascorbic acid, are commonly used in the food industry to protect from oxidative damage caused by reactive oxygen species, and indeed our results show that reducing agents prevent the enzyme inactivation happening under aerobic conditions in the presence of sulfite. The lack of LPMO products under these conditions shows that the positive effect of reductants is rather due to the scavenging of free radicals formed during oxidation of sulfite than to the promotion of LPMO activity. Obviously, utilization of large amounts of costly chemicals such as reductants is not economically feasible. Thus, the development of enzymes resistant to high sulfite concentrations or introduction of process steps for removing residual sulfite should be considered. Notably, further research on actual sulfite levels within the biorefinery is needed and so are studies on sulfite stability. The conditions used in this study, where we used high amounts of freshly added sodium sulfite, are not representative of the conditions in a real biorefinery.

It was known from before that lignosulfonates may have a positive effect on the saccharification of sulfite-pretreated lignocellulosic biomasses [34, 35]. These positive effects have been ascribed to the surfactant properties of LS [34] or to the formation of lignosulfonate–cellulase complexes [35], where both effects were thought to lead to reduced non-productive binding of cellulases to the substrate. In the present study, we observed that the positive effect of LS on hydrolysis efficiency was only present under aerobic conditions (Fig. 5a) and was linked to the promotion of LPMO activity (Fig. 5b). Interestingly, we also observed that LS reduced the negative effect of sulfite ions, though to a lesser extent than ascorbate. The exact mechanism of the stimulation of LPMO activity by LS addition is unknown and warrants further research. Notably, this effect could involve the interplay between (soluble) LS and residual lignin bound to the cellulose, which would be analogous to the interplay between solid and soluble lignin described by Westereng et al. [40].

## Conclusions

In this study, we have demonstrated efficient enzymatic conversion of sulfite-pretreated softwoods by Cellic^®^ CTec3 and we have demonstrated the importance of LPMOs in this process. We have also shown that, in order to obtain the high saccharification yields that are possible due to LPMO action, it is crucial that process conditions are adapted to the LPMOs, which need oxygen and reducing equivalents. The removal of residual sulfite from pretreatments is of major importance and both assessment of typical sulfite levels and methods to deal with these need further attention. Lignosulfonates have a potential to act as the electron donor for LPMOs, which could reduce the costs related to the addition of small molecule reductants in sulfite pretreatment-based biorefineries.

## Additional files

**Additional file 1: Figure S1.** Saccharification of pretreated Norway spruce in the absence (**a**, **b**) or presence (**c**, **d**) of sulfite under aerobic (*blue bars*) or anaerobic (*red bars*) conditions in the presence or absence of 1 mM gallic acid (+/− GA; *left panels*) or reduced glutathione (+/− RG; *right panels*). Enzymatic hydrolysis was carried out with Cellic® CTec3 at 8 mg/g glucan total protein loading in reaction mixtures containing 5% DM that were incubated in 50 mM sodium acetate pH 5.0, at 50 °C. Reactions with sulfite (*lower panels*) contained 1000 ppm of sulfite ions added as a sodium sulfite (12.5 mM). The data points represent the average value of three independent experiments with one technical replicate per experiment. The *error bars* represent standard deviations of the three independent experiments. The statistical significance of differences in the 48 hour saccharification yields was analyzed using two-way ANOVA with Tukey’s post hoc test (95% confidence interval), and is indicated as follows: *, p<0.05; **, p<0.01; ***, p<0.001.

**Additional file 2: Figure S2.** Saccharification of sulfite-pretreated Norway spruce without addition of l-cysteine hydrochloride monohydrate (0.025 % w/v) in the anaerobic reactions, in the absence (**a**, **b**) or presence (**c**, **d**) of sulfite under aerobic (*blue bars*) or anaerobic (*red bars*) conditions, in the presence or absence of 1 mM ascorbic acid (+/− AscA). The *left panels* show glucan conversion (as a percentage of theoretical glucan conversion) and the *right panels* show the concentration of Glc4gemGlc, at two time points. Enzymatic hydrolysis was carried out with Cellic® CTec3 at 8 mg/g glucan total protein loading in reaction mixtures containing 5% DM in 50 mM sodium acetate pH 5.0 that were incubated at 50 °C. Reactions with sulfite (*lower panels*) contained 1000 ppm of sulfite ions added as a sodium sulfite (12.5 mM). The data points represent the average value of three independent experiments with one technical replicate per experiment. The *error bars* represent standard deviations of the three independent experiments. The statistical significance of differences in the 48 hour saccharification yields was analyzed using two-way ANOVA with Tukey’s post hoc test (95% confidence interval), and is indicated as follows: *, p<0.05; **, p<0.01; ***, p<0.001.

**Additional file 3: Figure S3.** Saccharification of Avicel PH-101 in the presence of sulfite under aerobic (*blue bars*) or anaerobic (*red bars*) conditions, in the presence or absence of 1 mM ascorbic acid (+/− AscA). The *left panels* show glucan conversion (as a percentage of theoretical glucan conversion) and the *right panels* show concentration of Glc4gemGlc, at two time points. Enzymatic hydrolysis was carried out with Cellic® CTec3 (**a**, **b**) and Celluclast:Novozym 188 (mixed at 5:1 ratio, w/w) (**c**, **d**) at 8 mg/g glucan total protein loading in reaction mixtures containing 5 % DM in 50 mM sodium acetate pH 5.0 that were incubated at 50 °C. Data for similar reactions without sulfite are presented in Fig. 1. The data points represent the average value of three independent experiments with one technical replicate per experiment. The *error bars* represent standard deviations of the three independent experiments. The statistical significance of differences in the 48 hour saccharification yields was analyzed using two-way ANOVA with Tukey’s post hoc test (95% confidence interval), and is indicated as follows: *, p<0.05; **, p<0.01; ***, p<0.001.

## Abbreviations

AA: auxiliary activity
AscA: ascorbic acid
Glc4gemGlc: 4-hydroxy-β-d-xylo-hexopyranosyl-(1→4)-β-d-glucopyranosyl
HPAEC: high-performance anion exchange chromatography
HPLC: high-performance liquid chromatography
LPMO: lytic polysaccharide monooxygenase
LS: lignosulfonates
