# Supplementary figures and images for: Enzymatic degradation of sulfite-pulped softwoods and the role of LPMOs
Source: Biotechnol Biofuels. 2017 Jul 11;10:177. doi: 10.1186/s13068-017-0862-5 (PMC5504866; doi:10.1186/s13068-017-0862-5)

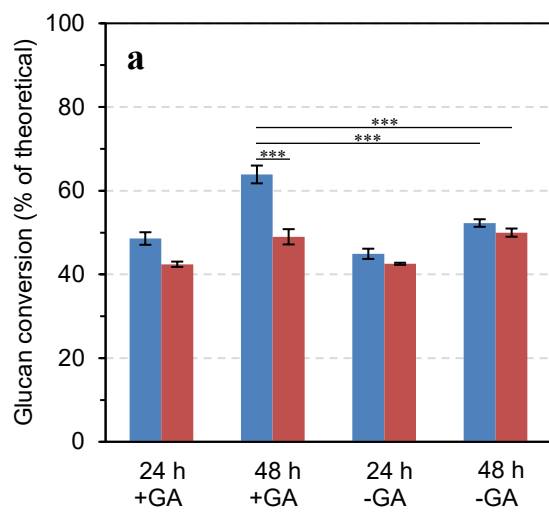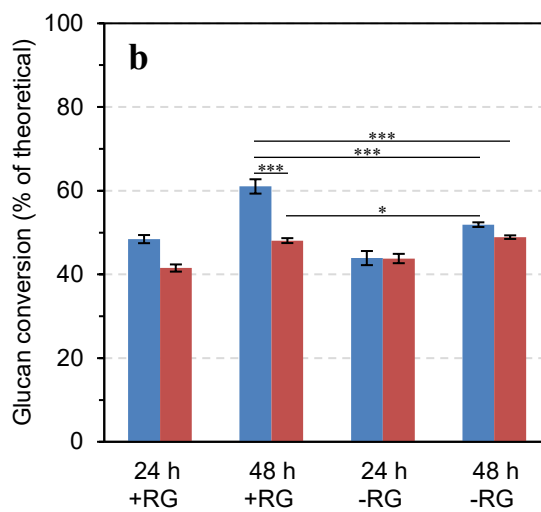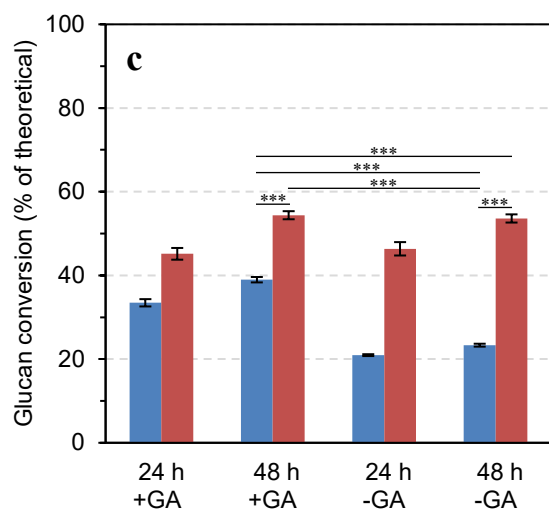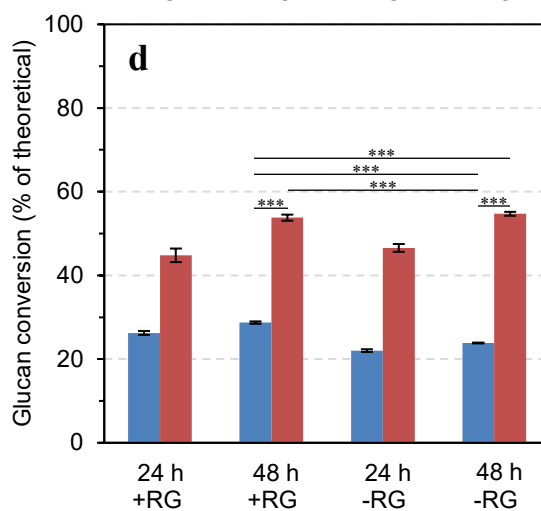

Supplement: Supplementary file 1 — Additional file 1: Figure S1. Saccharification of pretreated Norway spruce in the absence (a, b) or presence (c, d) of sulfite under aerobic (blue bars) or anaerobic (red bars) conditions in the presence or absence of 1 mM gallic acid (+/− GA; left panels) or reduced glutathione (+/− RG; right panels). Enzymatic hydrolysis was carried out with Cellic® CTec3 at 8 mg/g glucan total protein loading in reaction mixtures containing 5% DM that were incubated in 50 mM sodium acetate pH 5.0, at 50 °C. Reactions with sulfite (lower panels) contained 1000 ppm of sulfite ions added as a sodium sulfite (12.5 mM). The data points represent the average value of three independent experiments with one technical replicate per experiment. The error bars represent standard deviations of the three independent experiments. The statistical significance of differences in the 48 hour saccharification yields was analyzed using two-way ANOVA with Tukey’s post hoc test (95% confidence interval), and is indicated as follows: *, p<0.05; **, p<0.01; ***, p<0.001. [file 13068_2017_862_MOESM1_ESM.pdf]

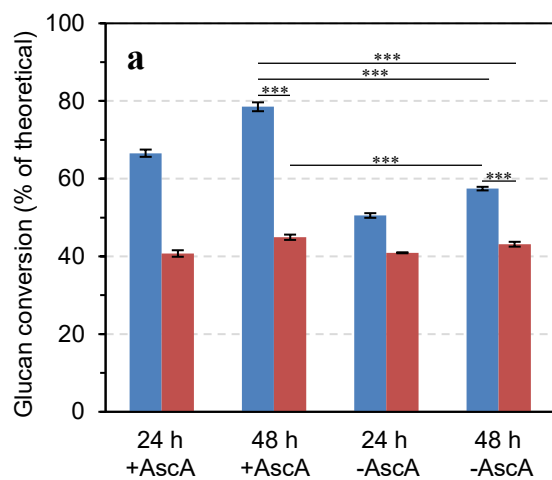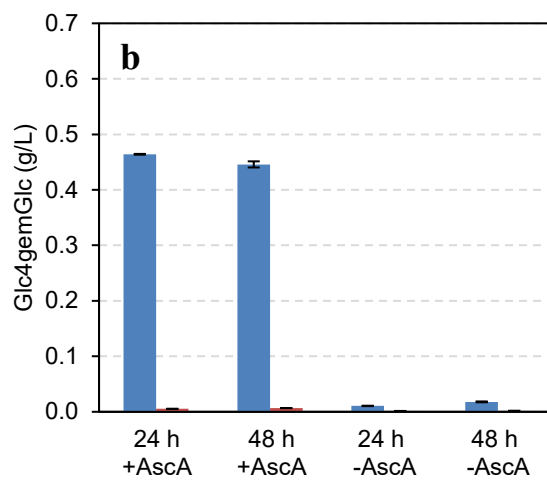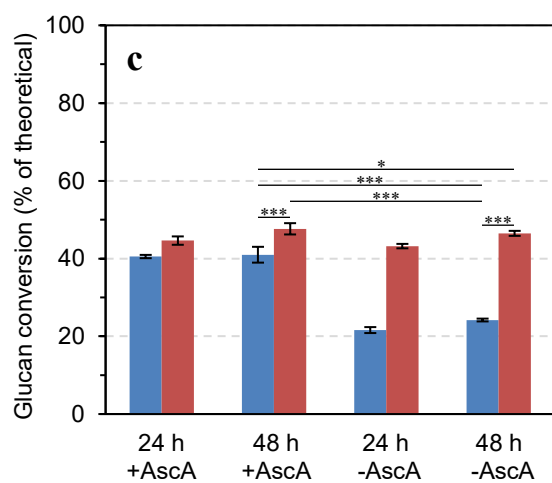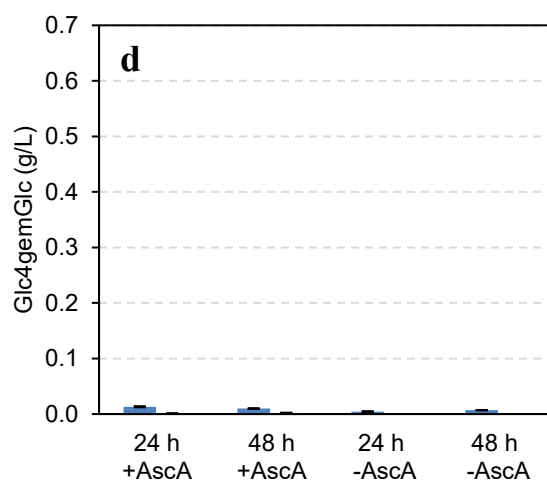

Supplement: Supplementary file 2 — Additional file 2: Figure S2. Saccharification of sulfite-pretreated Norway spruce without addition of l-cysteine hydrochloride monohydrate (0.025 % w/v) in the anaerobic reactions, in the absence (a, b) or presence (c, d) of sulfite under aerobic (blue bars) or anaerobic (red bars) conditions, in the presence or absence of 1 mM ascorbic acid (+/− AscA). The left panels show glucan conversion (as a percentage of theoretical glucan conversion) and the right panels show the concentration of Glc4gemGlc, at two time points. Enzymatic hydrolysis was carried out with Cellic® CTec3 at 8 mg/g glucan total protein loading in reaction mixtures containing 5% DM in 50 mM sodium acetate pH 5.0 that were incubated at 50 °C. Reactions with sulfite (lower panels) contained 1000 ppm of sulfite ions added as a sodium sulfite (12.5 mM). The data points represent the average value of three independent experiments with one technical replicate per experiment. The error bars represent standard deviations of the three independent experiments. The statistical significance of differences in the 48 hour saccharification yields was analyzed using two-way ANOVA with Tukey’s post hoc test (95% confidence interval), and is indicated as follows: *, p<0.05; **, p<0.01; ***, p<0.001. [file 13068_2017_862_MOESM2_ESM.pdf]

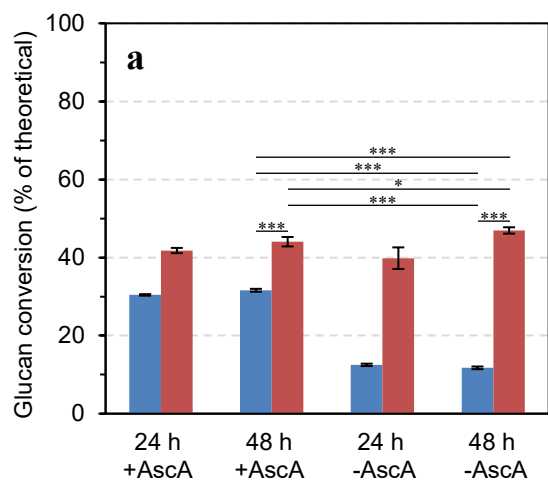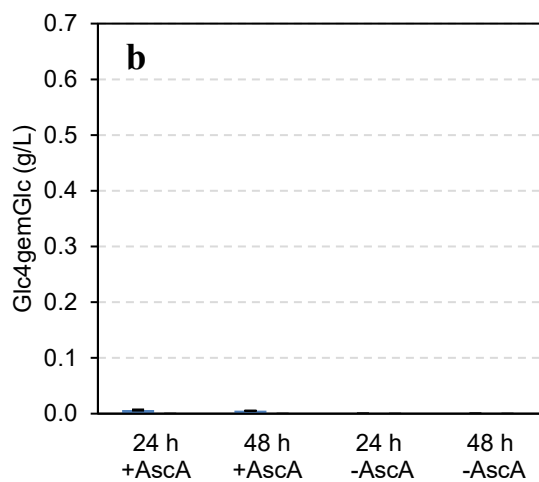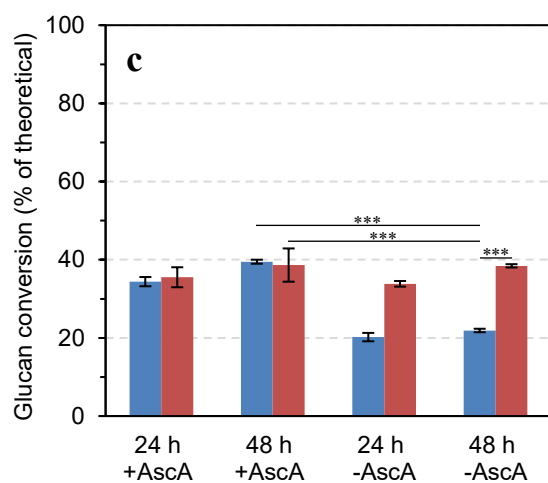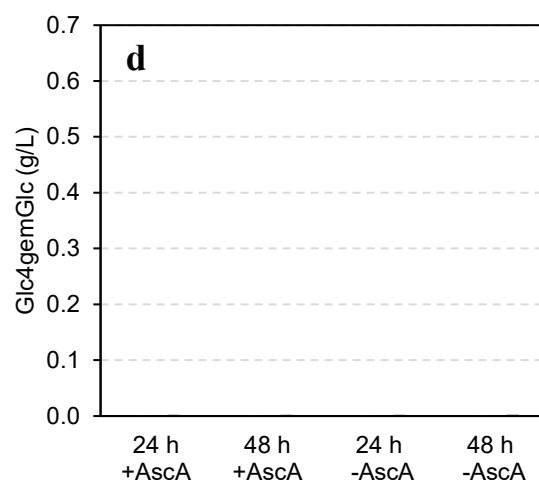

Supplement: Supplementary file 3 — Additional file 3: Figure S3. Saccharification of Avicel PH-101 in the presence of sulfite under aerobic (blue bars) or anaerobic (red bars) conditions, in the presence or absence of 1 mM ascorbic acid (+/− AscA). The left panels show glucan conversion (as a percentage of theoretical glucan conversion) and the right panels show concentration of Glc4gemGlc, at two time points. Enzymatic hydrolysis was carried out with Cellic® CTec3 (a, b) and Celluclast:Novozym 188 (mixed at 5:1 ratio, w/w) (c, d) at 8 mg/g glucan total protein loading in reaction mixtures containing 5 % DM in 50 mM sodium acetate pH 5.0 that were incubated at 50 °C. Data for similar reactions without sulfite are presented in Fig. 1. The data points represent the average value of three independent experiments with one technical replicate per experiment. The error bars represent standard deviations of the three independent experiments. The statistical significance of differences in the 48 hour saccharification yields was analyzed using two-way ANOVA with Tukey’s post hoc test (95% confidence interval), and is indicated as follows: *, p<0.05; **, p<0.01; ***, p<0.001. [file 13068_2017_862_MOESM3_ESM.pdf]
